# Supplementary material for: O-GlcNAc transferase inhibits visceral fat lipolysis and promotes diet-induced obesity
Source: Nat Commun. 2020 Jan 10;11:181. doi: 10.1038/s41467-019-13914-8 (PMC6954210; doi:10.1038/s41467-019-13914-8)
Supplement: Supplementary file 1 — Supplementary Information [file 41467_2019_13914_MOESM1_ESM.pdf]

Supplemental Information

Title: *O*-GlcNAc Transferase Inhibits Visceral Fat Lipolysis and Promotes Diet-induced Obesity

Authors: Yang et al.

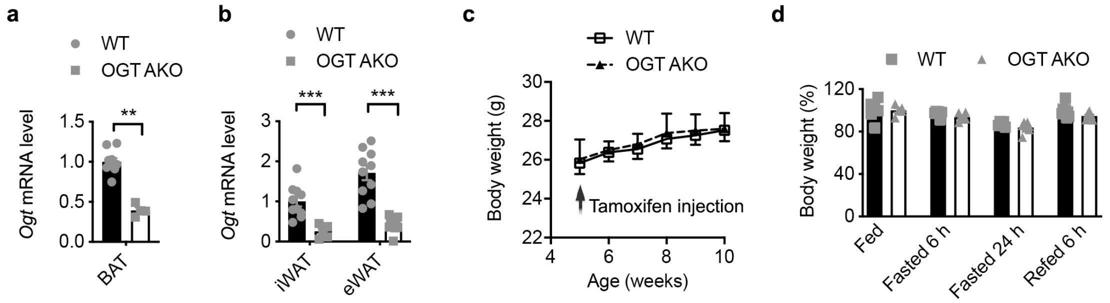

Supplementary Figure 1. Inducible deletion of adipose OGT does not affect body weight. **a, b** RT-PCR analysis of OGT mRNA levels in different fat depots from WT and OGT AKO mice (n = 10 WT, 4 KO for **a**; n = 8 WT, 6 or 10 KO for **b**); **c** Western blot analysis of OGT and β-actin in different fat depots from WT and OGT AKO mice (n = 11 WT, 4 KO); **d** Dynamic change of body weights of WT and OGT AKO mice during fasting/refeeding (n = 5-8/group). Data are presented as mean ± s.e.m. Statistical analysis: Student's t-test, ** $p < 0.01$, and *** $p < 0.001$. Source data are provided as a Source Data file.

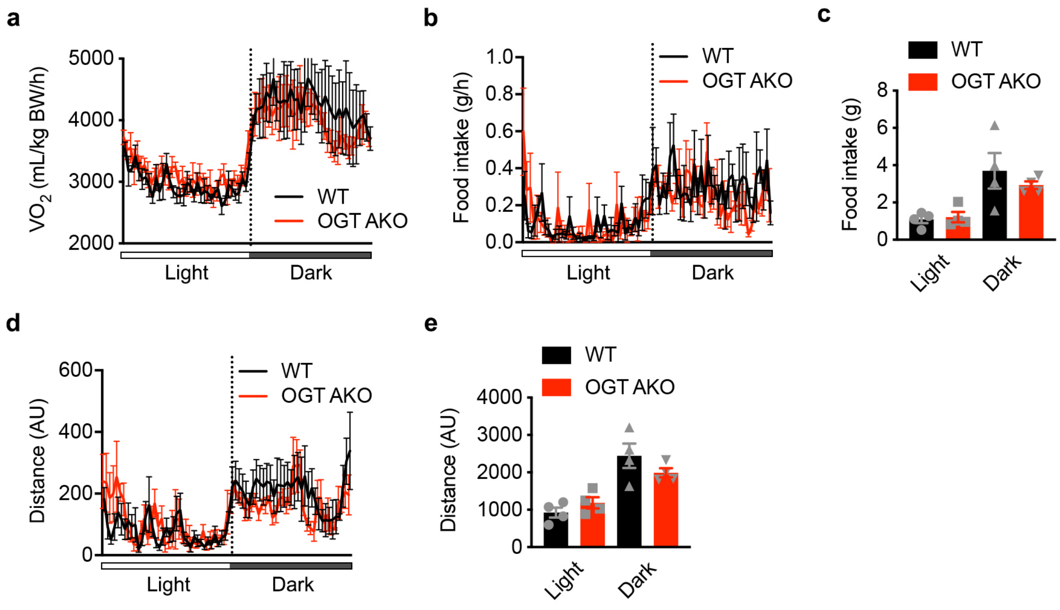

Supplementary Figure 2. OGT AKO mice have normal oxygen consumption rate, food intake and physical activity. Whole-body oxygen consumption rates **a**, food intake **b**, **c** and physical activities **d, e** of WT and OGT AKO mice (n = 4/group). Data are presented as mean ± s.e.m. Statistical analysis: Student’s t-test. Source data are provided as a Source Data file.

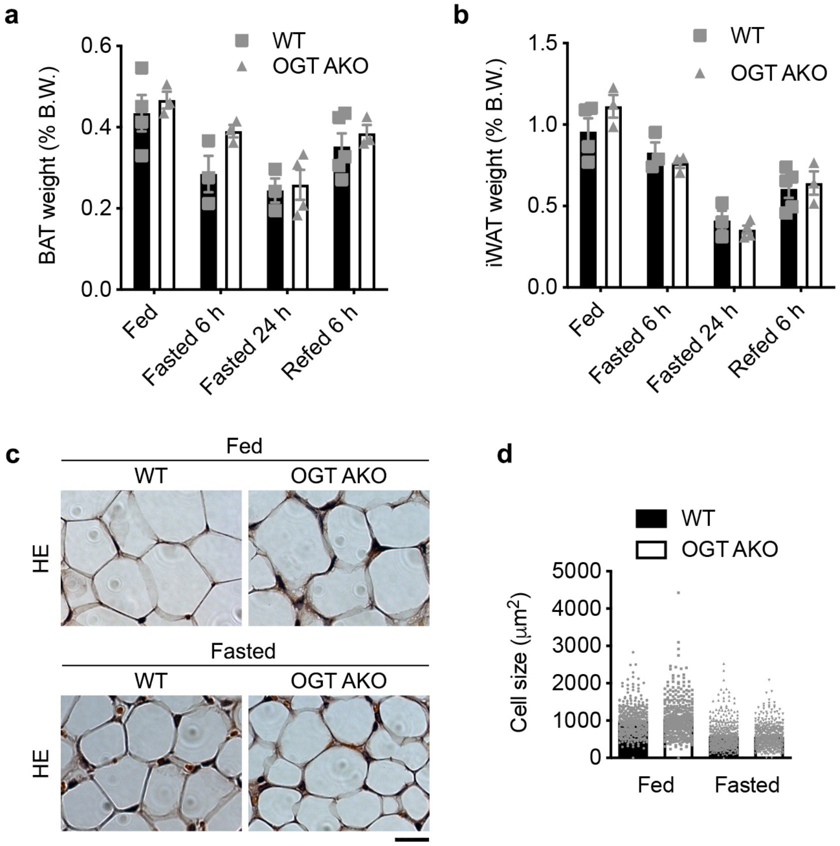

Supplementary Figure 3. Loss of adipose OGT does not affect the feeding/fasting response of BAT and iWAT. **a, b** Tissue weight of BAT and iWAT from WT and OGT AKO mice in fed, 6 h-fasted, 24 h-fasted, and 6 h-refed states (n = 3-5/group); **c** H&E staining of iWAT from WT and OGT AKO mice in fed and 24 h-fasted states, scale bar is 20 μm.; **d** Quantification of adipocyte size in iWAT shown in **c** (n > 350/group). Data are presented as mean ± s.e.m. Statistical analysis: Student's t-test. Source data are provided as a Source Data file.

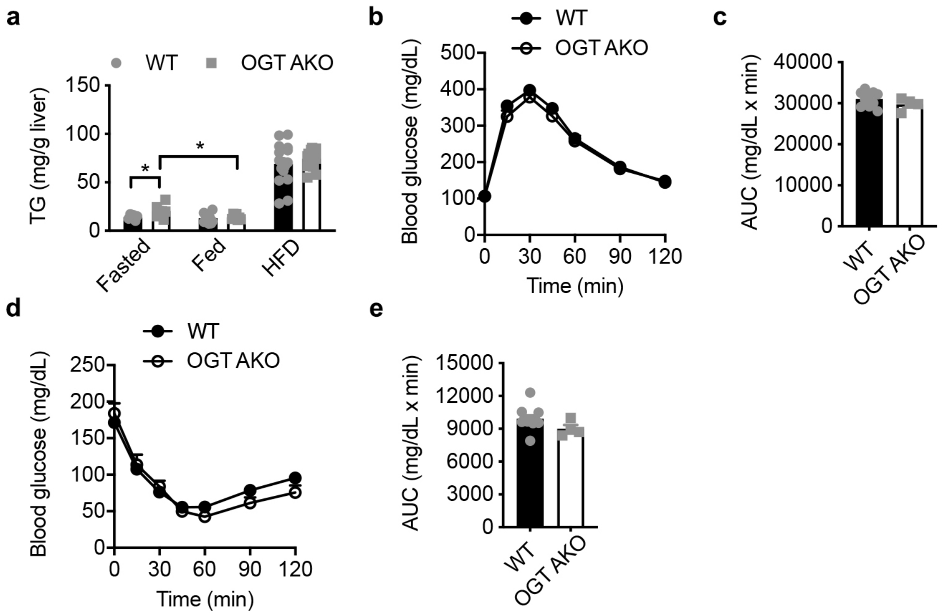

Supplementary Figure 4. Loss of adipose OGT does not affect whole-body glucose metabolism. **a** Hepatic triglyceride content evaluation of NC-fed WT and OGT AKO mice in fasted and refed states and HFD-fed WT and OGT AKO mice (n = 6-16/group); **b** Glucose tolerance test in WT and OGT AKO mice (n = 10 WT, 4 KO); **c** Area under curve analysis of glucose tolerance test; **d** Insulin tolerance test in WT and OGT AKO mice (n = 11 WT, 4 KO); **e** Area under curve analysis of insulin tolerance test. Data are presented as mean ± s.e.m. Statistical analysis: Student's t-test for **c**, **e** and ANOVA with Dunnett multiple comparisons for **a**, *$p < 0.05$. Source data are provided as a Source Data file.

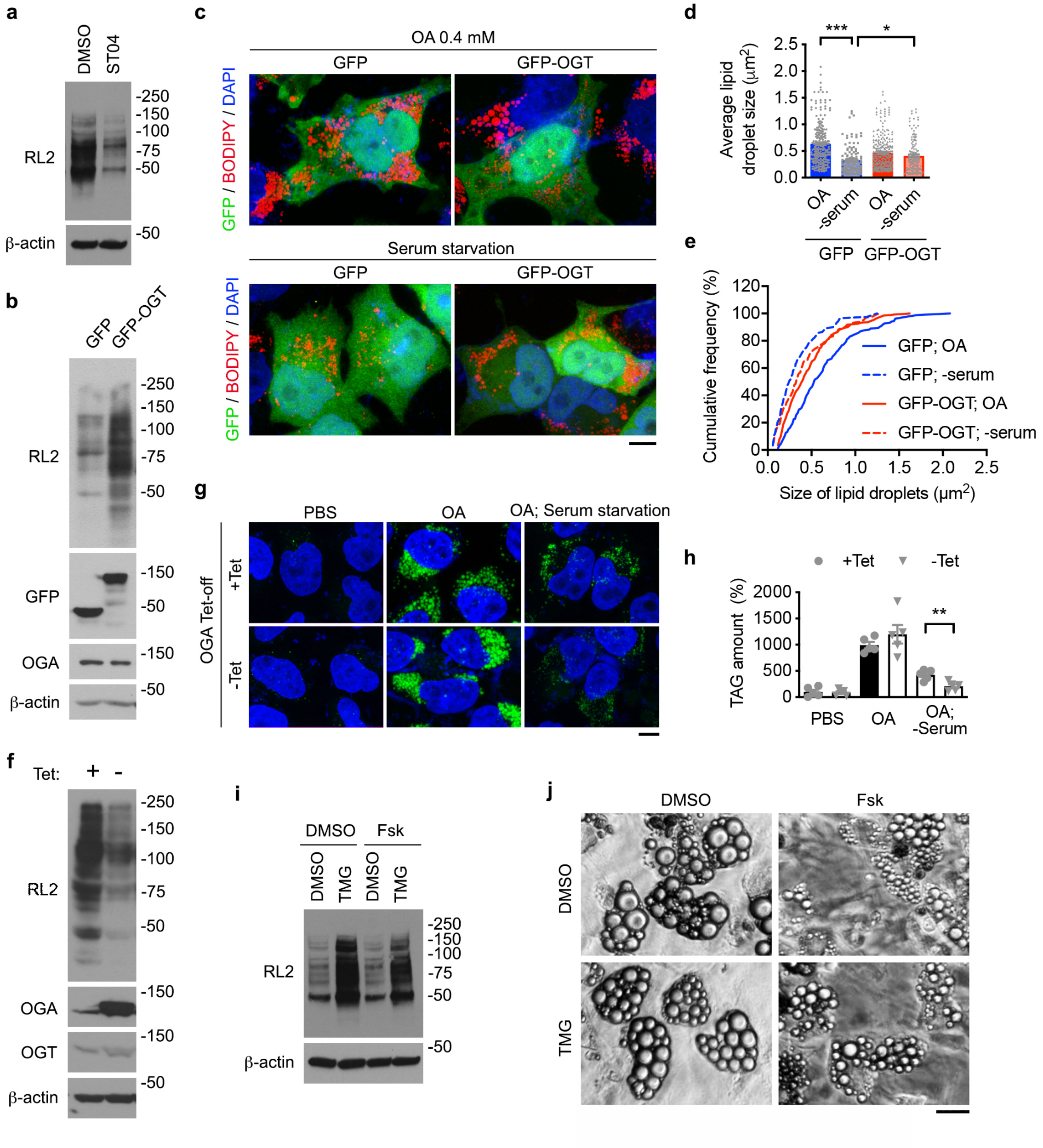

Supplementary Figure 5. *O*-GlcNAc signaling suppresses lipolysis in cultured cells. **a** Western blot analysis of overall *O*-GlcNAcylation and β-actin in differentiated C3H/10T1/2 cells treated with DMSO or ST04; RL2 recognizes *O*-GlcNAc modification on proteins; **b** Western blot analysis of overall *O*-GlcNAcylation, GFP, OGA and β-actin in HeLa cells overexpressing GFP or GFP-OGT; **c** Fluorescence images of lipid droplets (BODIPY 493/503, red) and nuclei (DAPI, blue) in HeLa cells overexpressing GFP or GFP-OGT (green), scale bar is 5 μm.; **d, e** Quantification of lipid droplet size in HeLa cells shown in **c** (n > 100/group); **f** Western blot analysis of overall *O*-

GlcNAcylation, OGA, OGT and β-actin in control (+Tet) and OGA-overexpressing (-Tet) HeLa cells; The overexpression of OGA is controlled by a Tet-off system; **g** Fluorescence images of lipid droplets (BODIPY 493/503, green) and nuclei (DAPI, blue) in control (+Tet) and OGA-overexpressing (-Tet) HeLa cells (n = 5/group), scale bar is 5 µm.; **h** Triglyceride content evaluation of HeLa cells shown in **g**; **i** Western blot analysis of overall *O*-GlcNAcylation and β-actin in *in vitro* cultured adipocytes pretreated with DMSO or OGA inhibitor TMG, and incubated with DMSO or Fsk, **j** Bright field microscopy of *in vitro* cultured adipocytes pretreated with DMSO or TMG, and incubated with DMSO or Fsk, scale bar is 20 µm. Data are presented as mean ± s.e.m. Statistical analysis: ANOVA with Dunnett multiple comparisons for **d** and Student's t-test for **h**, *$p < 0.05$, **$p < 0.01$, and ***$p < 0.001$. Source data are provided as a Source Data file.

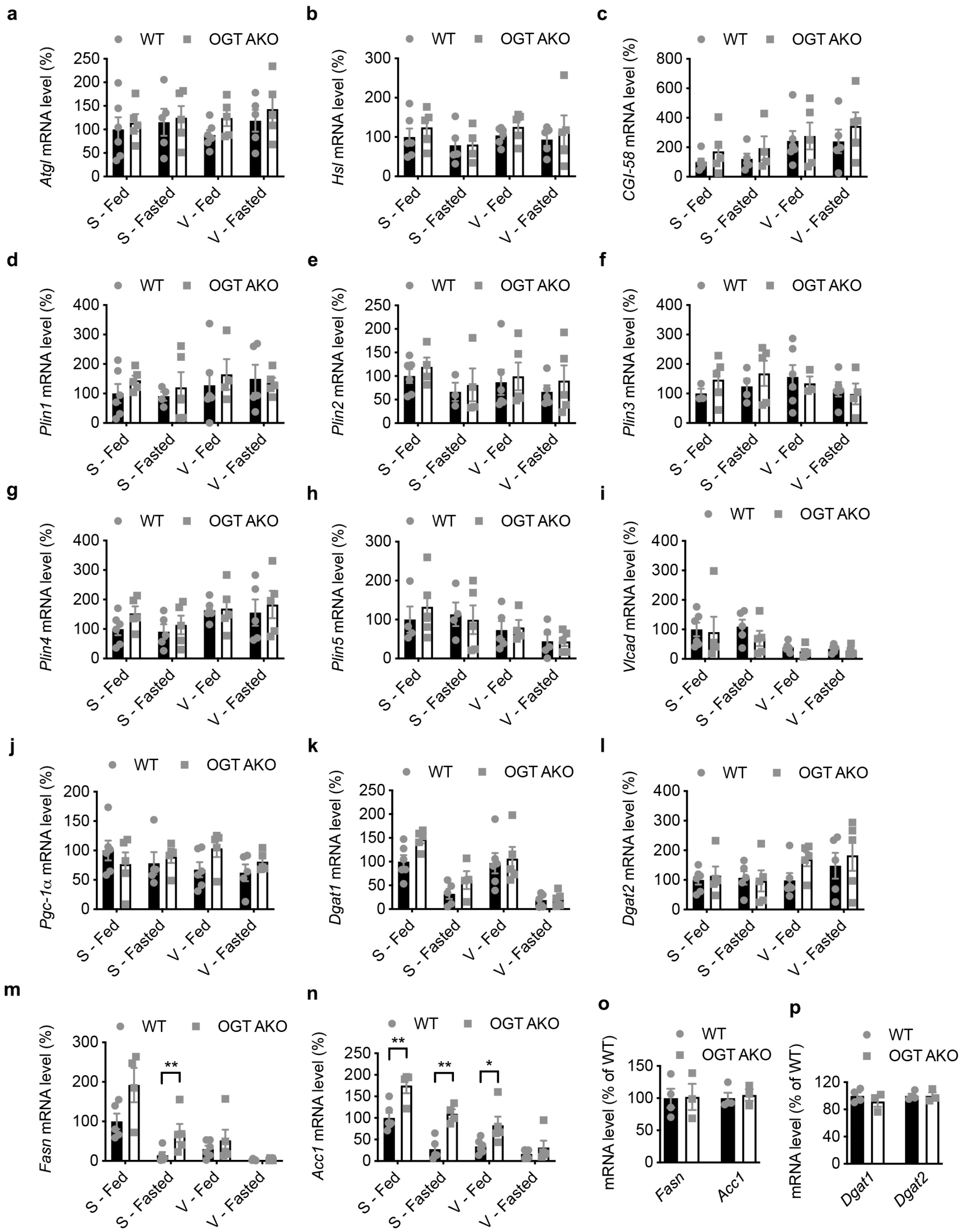

Supplementary Figure 6. Effect of OGT depletion on the transcriptional regulation of lipid metabolism. **a-h** RT-PCR analysis of mRNA levels of lipolytic enzymes ATGL and HSL, and lipolysis-regulating proteins CGI-58 and PLIN family proteins in iWAT (S) and eWAT (V) from WT

and OGT AKO mice in fed and fasted states (n = 5-6 WT, 4-5 KO); **i-n** RT-PCR analysis of mRNA levels of enzymes involved in beta-oxidation and lipogenesis including VLCAD, PGC-1α, DGAT1, DGAT2, FASN and ACC1 in iWAT (S) and eWAT (V) from WT and OGT AKO mice in fed and fasted states (n = 5-6 WT, 4-5 KO); **o, p** RT-PCR analysis of mRNA levels of key lipogenic enzymes including DGAT1, DGAT2, FASN and ACC1 in eWAT from WT and OGT AKO mice in refed status (n = 4 WT, 3 KO). Data are presented as mean ± s.e.m. Statistical analysis: Student's t-test, *$p < 0.05$, and **$p < 0.01$. Source data are provided as a Source Data file.

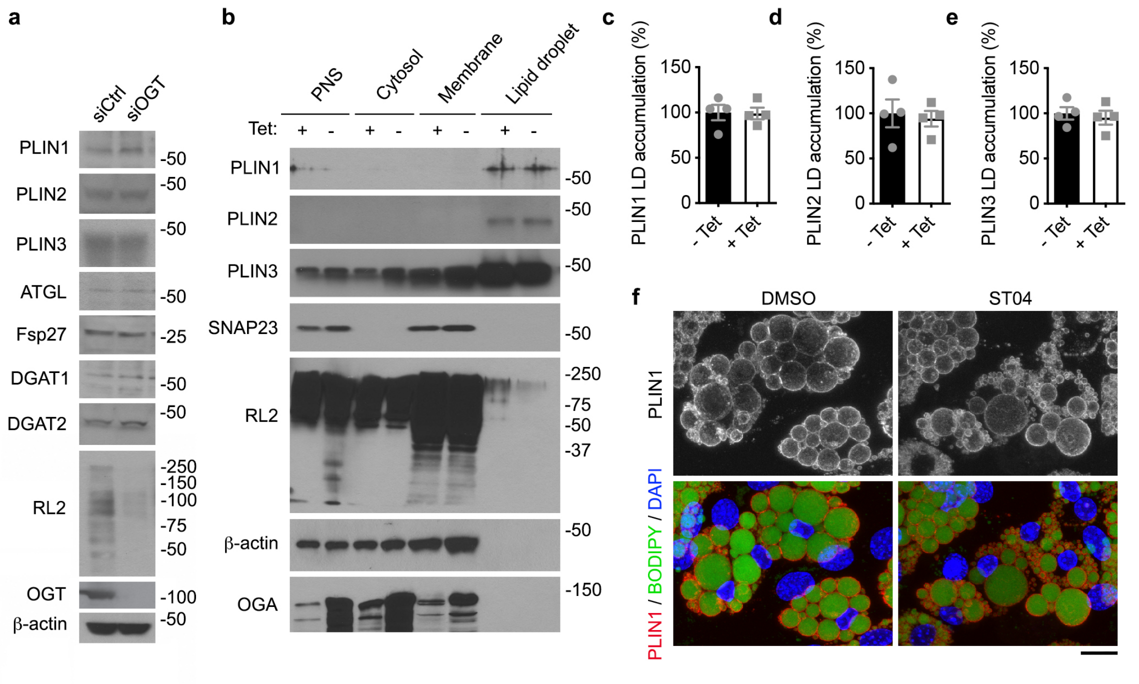

Supplementary Figure 7. Inhibition of *O*-GlcNAc signaling does not affect the expression and the lipid droplet accumulation of PLIN proteins. **a** Western blot analysis of PLIN family protein PLIN1~3, lipolytic enzyme ATGL, lipolysis regulator Fsp27, lipogenic enzymes DGAT1 and DGAT2, overall *O*-GlcNAcylation, OGT, and β-actin in control and OGT siRNA-treated HeLa cells; **b** Representative Western blots of PLIN1~3, SNAP23, *O*-GlcNAcylation, β-actin, and OGA in subcellular fractions of control (+Tet) and OGA-overexpressing (-Tet) HeLa cells; Experiments were repeated three times; **c-e** Quantification of lipid droplet localization of PLIN1~3 in HeLa cells determined in **b** (n = 4/group); **f** Immunofluorescence staining of PLIN1 (red) in HeLa cells treated with DMSO or OGT inhibitor ST04; lipid droplets were stained with BODIPY 493/503 (green) and nuclei were stained with DAPI (blue), scale bar is 5 µm. Data are presented as mean ± s.e.m. Statistical analysis: Student's t-test. Source data are provided as a Source Data file.

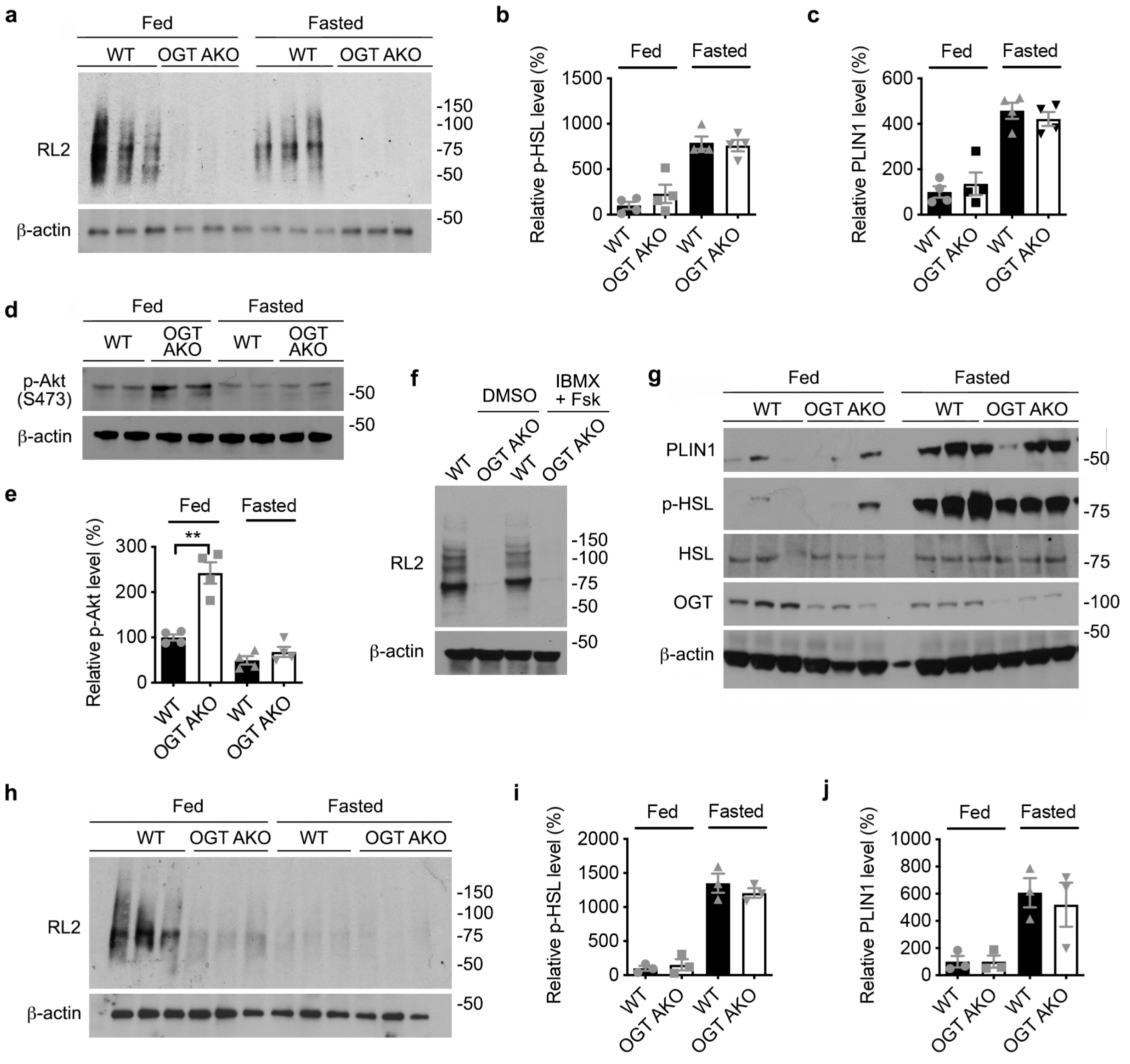

Supplementary Figure 8. Effects of OGT depletion on overall *O*-GlcNAcylation, PLIN1 expression, Akt phosphorylation and HSL phosphorylation in visceral and subcutaneous fat. **a** Western blot analysis of *O*-GlcNAcylation and β-actin in eWAT of fed and 6 h-fasted WT and OGT AKO mice; **b, c** Quantification of levels of p-HSL and PLIN1 in eWAT of fed and 6 h-fasted WT and OGT AKO mice (n = 4/group); **d, e** Representative Western blots and quantification of Akt phosphorylation at serine 473 (S473) in eWAT of fed and 6h-fasted WT and OGT AKO mice (n = 4/group); **f** Western blot analysis of *O*-GlcNAcylation and β-actin in primary adipocytes differentiated *in vitro* from the WT and OGT AKO eWAT SVFs; IBMX and Fsk were used to stimulate lipolysis; **g, h** Western blot analysis of PLIN1, p-HSL, HSL, OGT, *O*-GlcNAcylation and β-actin in iWAT of fed and 6 h-fasted WT and OGT AKO mice; **i, j** Quantification of levels of p-HSL and PLIN1 in iWAT of fed and 6 h-fasted WT and OGT AKO mice (n = 3/group). Data are presented as mean ± s.e.m. Statistical analysis: Student's t-test, $^{**}p < 0.01$. Source data are provided as a Source Data file.

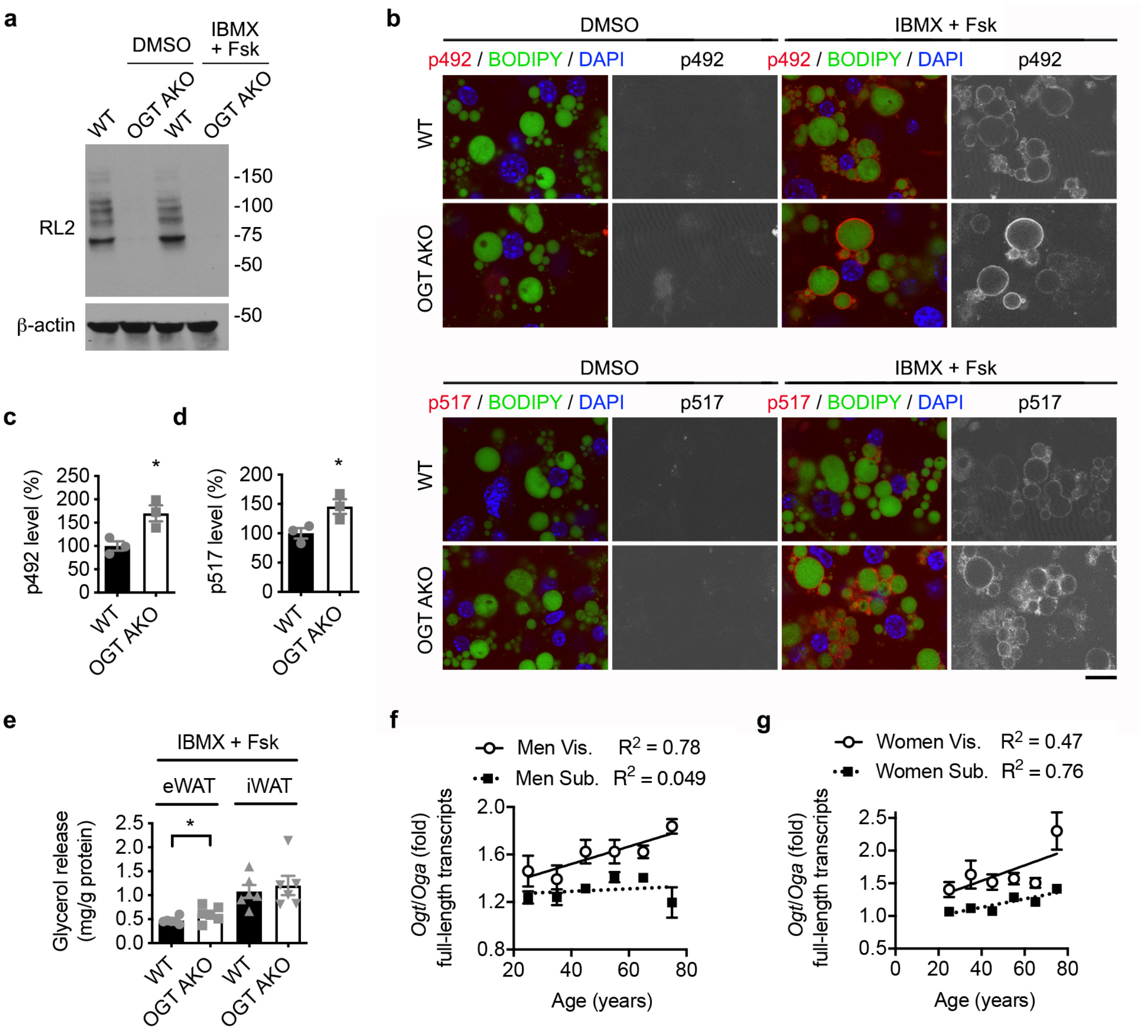

Supplementary Figure 9. Conserved OGT-regulated PLIN1 phosphorylation, but differentially regulated *O*-GlcNAc signaling and lipolysis in subcutaneous fat. **a** Western blot analysis of *O*-GlcNAcylation and β-actin in primary adipocytes differentiated *in vitro* from the WT and OGT AKO iWAT SVFs; IBMX and Fsk were used to stimulate lipolysis; **b-d** Immunofluorescence staining and quantification of PLIN1 phosphorylation at serine 492 (p492) and serine 517 (p517) in primary adipocytes differentiated *in vitro* from the WT and OGT AKO iWAT SVFs (n = 3/group); lipid droplets were stained with BODIPY 493/503 (green) and nuclei were stained with DAPI (blue), scale bar is 20 µm.; **e** IBMX/Fsk-stimulated lipolysis measured by glycerol released from explants of eWAT and iWAT from WT and OGT AKO mice (n = 6/group); **f, g** Ratios of OGT/OGA mRNA levels (full-length transcripts) in subcutaneous fat (Sub.) and visceral fat (Vis.) from Men and Women in different age groups (n = 6 for ~75 year old Men Vis., n > 14 for other groups in **f**; n = 3 for ~75 year old Women Vis., n = 7 for ~75 year old Women Sub., n > 10 for other groups in **g**); Original data was from the Genotype-Tissue Expression (GTEx) project. Data are presented as mean ± s.e.m. Statistical analysis: Student's t-test, *$p < 0.05$. Source data are provided as a Source Data file.

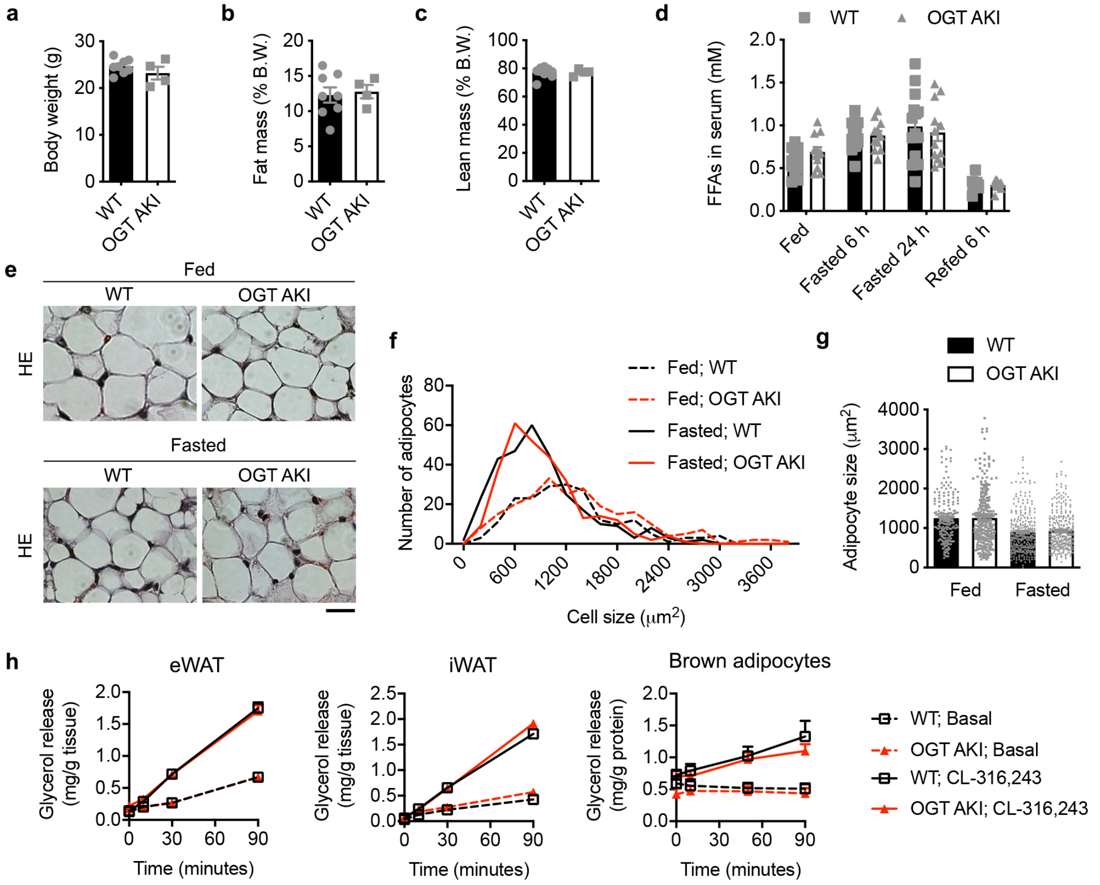

Supplementary Figure 10. Overexpression of adipose OGT does not affect fasting-induced lipolysis in NC-fed mice. **a-c** Body weight and body composition of WT and OGT AKI mice fed on NC (n = 8 WT, 4 KI); **d** Dynamic changes in serum FFA level in WT and OGT AKI mice during fasting/refeeding (n = 14 WT, 11-12 KI); **e** H&E staining of eWAT from WT and OGT AKI mice in fed and fasted states, scale bar is 20 µm.; **f, g** Quantification of adipocyte size in eWAT shown in **e** (n > 190/group); **h** Basal and stimulated (10 µM CL-316,243) lipolysis measured by glycerol released from explants of eWAT and iWAT and isolated brown adipocytes from NC-fed WT and OGT AKI mice (n = 3-5/group). Data are presented as mean ± s.e.m. Statistical analysis: Student's t-test. Source data are provided as a Source Data file.

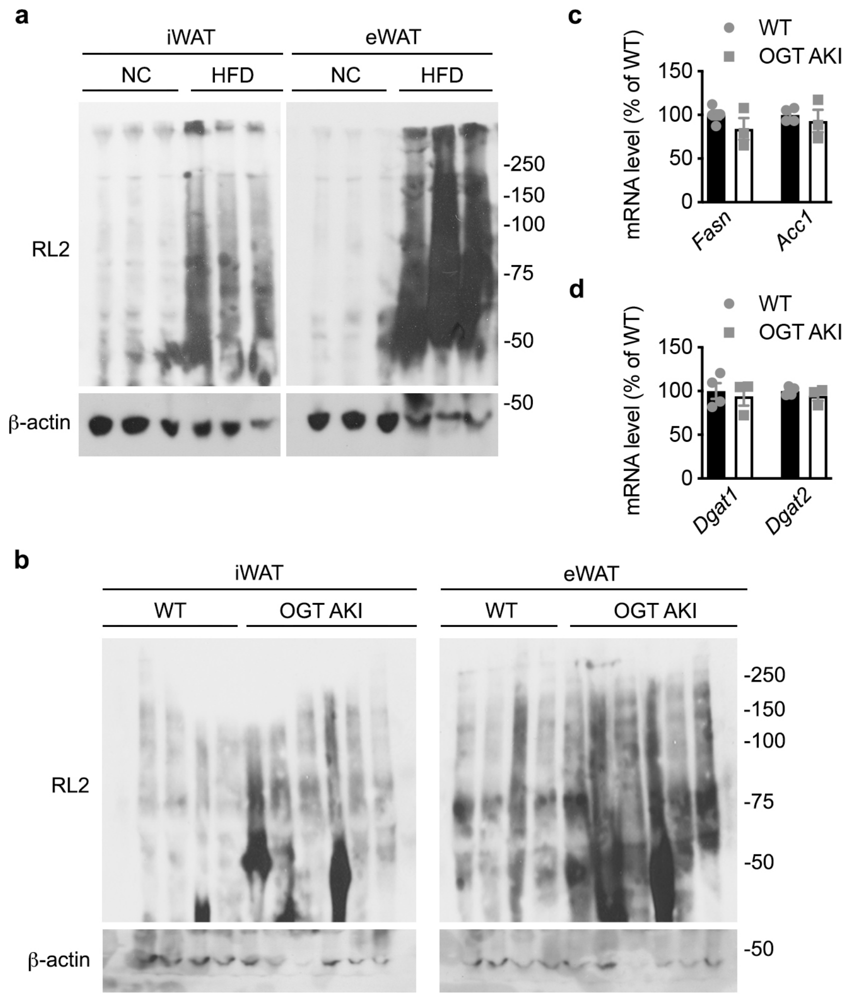

Supplementary Figure 11. Examination of overall *O*-GlcNAcylation level and lipogenic gene expression in iWAT and/or eWAT of HFD-fed WT and OGT AKI mice. **a** Western blot analysis of overall *O*-GlcNAcylation and β-actin in iWAT and eWAT from NC-fed and HFD-fed WT mice; **b** Western blot analysis of overall *O*-GlcNAcylation and β-actin in iWAT and eWAT from HFD-fed WT and OGT AKI mice; **c, d** RT-PCR analysis of mRNA levels of key lipogenic enzymes including FASN, ACC1, DGAT1 and DGAT2 in eWAT from HFD-fed WT and OGT AKI mice (n = 4 WT, 3 KI). Data are presented as mean ± s.e.m. Statistical analysis: Student's t-test. Source data are provided as a Source Data file.

Supplementary Table 1. List of primers

Gene	Forward	Reverse
Ogt	AAGAGGCACGCATTTTTGAC	ATGGGGTTGCAGTTCGATAG
Atgl	TAATGTTGGCACCTGCTTCA	CCACTCACATCTACGGAGCC
Hsl	GGAGAGAGTCTGCAGGAACG	CCTGCAAGAGTATGTCACGC
CGI-58	TGACAGTGATGCGGAAGAAG	AGATCTGGTCGCTCAGGAAA
Plin1	GGCCAACACTCTTTCTCGACACA	ACTCTTCTTCCTCCTCCTCGTCGT
Plin2	CAGTGGAGTAGATAATGCCATCAC	GCTTCTGAACCATATCAAATCCTT
Plin3	GTGTGGGACAGATGGTGAT	AAGTAGTTCTGCTCCTGTCG
Plin4	GTGTCCACCAACTCACAGATG	GGACCATTCCTTTTGCAGCAT
Plin5	CTTCCTGCCCATGACTGAGG	GACCCCAGACGCACAAAGTAG
Vlcad	CCGGTTCTTTGAGGAAGTGAA	AGTGTCGTCCTCCACCTTCTC
Pgc-1α	AACCACACCCACAGGATCAGA	TCTTCGCTTTATTGCTCCATGA
Dgat1	GAGGCCTCTCTGCCCCTATG	GCCCCTGGACAACACAGACT
Dgat2	CCGCAAAGGCTTTGTGAAG	GGAATAAGTGGGAACCAGATCA
Fasn	GAGGACACTCAAGTGGCTGA	GTGAGGTTGCTGTCGTCTGT
Acc1	GAAGCCACAGTGAAATCTCG	GATGGTTTGGCCTTTCACAT
