# Supplementary material for: O-GlcNAc transferase inhibits visceral fat lipolysis and promotes diet-induced obesity
Source: Nat Commun. 2020 Jan 10;11:181. doi: 10.1038/s41467-019-13914-8 (PMC6954210; doi:10.1038/s41467-019-13914-8)
Supplement: Supplementary file 3 — Reporting summary [file 41467_2019_13914_MOESM3_ESM.pdf]

nature research

Corresponding author(s): Xiaoyong Yang

Last updated by author(s): Sep 8, 2019

Reporting Summary

Nature Research wishes to improve the reproducibility of the work that we publish. This form provides structure for consistency and transparency in reporting. For further information on Nature Research policies, see Authors & Referees and the Editorial Policy Checklist.

Statistics

For all statistical analyses, confirm that the following items are present in the figure legend, table legend, main text, or Methods section.

n/a	Confirmed	
☐	☒	The exact sample size (*n*) for each experimental group/condition, given as a discrete number and unit of measurement
☐	☒	A statement on whether measurements were taken from distinct samples or whether the same sample was measured repeatedly
☐	☒	The statistical test(s) used AND whether they are one- or two-sided<br>*Only common tests should be described solely by name; describe more complex techniques in the Methods section.*
☐	☒	A description of all covariates tested
☒	☐	A description of any assumptions or corrections, such as tests of normality and adjustment for multiple comparisons
☐	☒	A full description of the statistical parameters including central tendency (e.g. means) or other basic estimates (e.g. regression coefficient) AND variation (e.g. standard deviation) or associated estimates of uncertainty (e.g. confidence intervals)
☐	☒	For null hypothesis testing, the test statistic (e.g. *F*, *t*, *r*) with confidence intervals, effect sizes, degrees of freedom and *P* value noted<br>*Give P values as exact values whenever suitable.*
☒	☐	For Bayesian analysis, information on the choice of priors and Markov chain Monte Carlo settings
☒	☐	For hierarchical and complex designs, identification of the appropriate level for tests and full reporting of outcomes
☒	☐	Estimates of effect sizes (e.g. Cohen's *d*, Pearson's *r*), indicating how they were calculated

Our web collection on statistics for biologists contains articles on many of the points above.

Software and code

Policy information about availability of computer code

Data collection	No custom algorithms or new softwares were used.
Data analysis	No custom algorithms or new softwares were used.

For manuscripts utilizing custom algorithms or software that are central to the research but not yet described in published literature, software must be made available to editors/reviewers. We strongly encourage code deposition in a community repository (e.g. GitHub). See the Nature Research guidelines for submitting code & software for further information.

Data

Policy information about availability of data

All manuscripts must include a data availability statement. This statement should provide the following information, where applicable:

- Accession codes, unique identifiers, or web links for publicly available datasets
- A list of figures that have associated raw data
- A description of any restrictions on data availability

The source data underlying most figures are provided as a Source Data file. Other datasets generated and/or analyzed in the current study are available from the corresponding author upon reasonable request.

Field-specific reporting

Please select the one below that is the best fit for your research. If you are not sure, read the appropriate sections before making your selection.

☒ Life sciences ☐ Behavioural & social sciences ☐ Ecological, evolutionary & environmental sciences

For a reference copy of the document with all sections, see nature.com/documents/nr-reporting-summary-flat.pdf

Life sciences study design

All studies must disclose on these points even when the disclosure is negative.

Sample size	No sample size calculation in this study
Data exclusions	No data exclusion in this study
Replication	All experiments were repeated at least three times and similar results were observed.
Randomization	No randomization in this study
Blinding	No blinding in this study

Reporting for specific materials, systems and methods

We require information from authors about some types of materials, experimental systems and methods used in many studies. Here, indicate whether each material, system or method listed is relevant to your study. If you are not sure if a list item applies to your research, read the appropriate section before selecting a response.

Materials & experimental systems

n/a	Involved in the study	
☐	☒	Antibodies
☐	☒	Eukaryotic cell lines
☒	☐	Palaeontology
☐	☒	Animals and other organisms
☒	☐	Human research participants
☒	☐	Clinical data

Methods

n/a	Involved in the study	
☒	☐	ChIP-seq
☒	☐	Flow cytometry
☒	☐	MRI-based neuroimaging

Antibodies

Antibodies used	Antibodies against OGT (ab96718), OGA (MGEA5, ab124807), O-GlcNAc (RL2, ab2739), PLIN1 (ab3526), SNAP23 (ab3340), ATGL (ab99532), DGAT1 (11561-1-AP), Fsp27 (CIDE C, ab77115), and p-Ser (Phosphoserine, PSR-45) (Abcam); p492 phosphorylated PLIN1 (4855) and p517 phosphorylated PLIN1 (4856) (Vala Sciences); HA (H3663) (Sigma-Aldrich); CGI-58 (ABHD5, 12201-1-AP), PLIN2 (15294-1-AP), and PLIN3 (TIP47, 10694-1-AP) (Proteintech); p563-HSL (4139), phospho-Akt (Ser473, 9271), and Akt (9272) (Cell Signaling Technology); Myc (sc-40), DGAT2 (sc-66859), and β-actin (sc-8432) (Santa Cruz Biotechnology) were purchased from the indicated sources. Horseradish peroxidase-conjugated secondary antibodies were from Santa Cruz Biotechnology. Alexa Fluor 594-conjugated secondary antibodies were obtained from Thermo Fisher Scientific.
Validation	All primary antibodies are commercially available and validation results can be found at manufacturer's websites.

Eukaryotic cell lines

Policy information about cell lines

Cell line source(s)	C3H/10T1/2, HeLa, and 293T cells were from the American Type Culture Collection (ATCC). OGA-Tet off HeLa stable cell line was established in our laboratory.
Authentication	Cell lines were not authenticated.
Mycoplasma contamination	C3H/10T1/2, HeLa, and 293T cells were tested mycoplasma-free.
Commonly misidentified lines (See ICLAC register)	No commonly misidentified lines in this study

Animals and other organisms

Policy information about studies involving animals; ARRIVE guidelines recommended for reporting animal research

Laboratory animals	Ogt-flox, Adipoq-CreER and Rosa26-STOPflox-rOGT mice in the C57BL/6 background were used. Male mice were used unless otherwise stated. Experiments were performed in 4~12 weeks old mice used unless otherwise stated.

Wild animals	No wild animals were used.
Field-collected samples	No field-collected samples were used.
Ethics oversight	All relevant ethical regulations for animal testing and research have been complied with. All animal studies received ethical approval from Yale University's Institutional Animal Care and Use Committee.

Note that full information on the approval of the study protocol must also be provided in the manuscript.
